# Emerging Viruses without Borders: The Wuhan Coronavirus

**DOI:** 10.3390/v12020130

**Published:** 2020-01-22

**Authors:** Shan-Lu Liu, Linda Saif

**Affiliations:** 1 Center for Retrovirus Research, The Ohio State University, Columbus, OH 43210, USA; 2 Department of Veterinary Biosciences, The Ohio State University, Columbus, OH 43210, USA; 3 Department of Microbial Infection and Immunity, The Ohio State University, Columbus, OH 43210, USA; 4 Viruses and Emerging Pathogens Program, Infectious Diseases Institute, The Ohio State University, Columbus, OH 43210, USA; saif.2@osu.edu; 5 Food Animal Health Research Program, The Ohio Agricultural Research and Development Center, College of Food, Agriculture and Environmental Sciences, The Ohio State University, Wooster, OH 43210, USA; 6 Department of Veterinary Preventive Medicine, College of Veterinary Medicine, The Ohio State University, Wooster, OH 43210, USA

## Abstract

The recently emerged coronavirus in Wuhan, China has claimed at least six lives as of January 22 and infected hundreds if not thousands of individuals. The situation has drawn international attention, including from the virology community. We applaud the rapid release to the public of the genome sequence of the new virus by Chinese virologists, but we also believe that increased transparency on disease reporting and data sharing with international colleagues are crucial for curbing the spread of this newly emerging virus to other parts of the world.

It is now clear that the mysterious respiratory illness in Wuhan is caused by a new type of coronavirus distantly related to the SARS coronavirus (SARS-CoV) [1,2]. We applaud the rapid release to the public of the genome sequence of the new virus by Chinese virologists [3], as this represents an important first step in curbing the spread of the new virus to other parts of the world.

Viruses emerge and re-emerge globally without consideration for borders. In the recent past, we have witnessed outbreaks of SARS, Ebola, Chikungunya, and Zika [4]. With each new outbreak, lives are lost, and the world is placed on high alert. Lessons have been learned from the initial coverup and misidentification of the SARS pathogen in 2003 [5,6], and the recent slow response of the World Health Organization to the 2014–2015 Ebola outbreak [7]. From this perspective, it is understandable that Chinese scientists cautiously released the identity and genome sequence of this new virus [3]. However, transparency on disease reporting to the public and data sharing with international colleagues must continue. With an increasing number of new cases of infection by the new coronavirus reported in China and neighboring countries, such as Japan and Thailand [8], human-to-human transmission should be thoroughly investigated.

The international virology community, which includes many Chinese American virologists residing in North America, is concerned about this virus. It is imperative that animal reservoirs that transmitted the virus to humans be identified as quickly as possible to aid in control of this disease. It is thus urgent that the results of animal testing from the seafood market in Wuhan, where the virus was initially isolated, be released as soon as possible. This is particularly crucial given the rapidly approaching Lunar Chinese New Year, which will take place on 25 January 2020, during which tens of millions of people will travel, and large amounts of animal meats, some of which may contain this virus, will be consumed. It is noteworthy that recently the local health administration in Wuhan issued guidance regarding the prevention and treatment of the pneumonia caused by this coronavirus [9]. It is also encouraging that the World Health Organization has issued detailed guidance on the clinical management of the disease in relation to this outbreak [10].

Viruses spread irrespective of borders—they jump from animals to humans, and they move from one country to another. Controlling the spread of emerging and re-emerging viruses requires international efforts and collaboration. We anticipate that scientific data and reagents will be shared publicly and fairly, and most importantly, that the scientific collaborations between the US and China, including the study of emerging viruses and infectious diseases, will continue unabated despite some turmoil in other aspects of the US-China relationship.
